# 

**Published:** 1805-07-01

**Authors:** 


### MEDICAL

AND

### PHYSICAL

### *JOURNAL.*

CONDUCTED BY

T. BRADLEY, M.D.

R. BATTY, M.D.

AND

J. ARNEMAN, M.D.

VOL. XIV.

FROM JUNE TO DECEMBER, 1805.

5120

*LONDON:*

PRINTED FOR RICHARD PHILLIPS, NO. 6, BRIDGE-STREET, BLACKFRIARS.

[By William Thorne, Red Lion Court, Fleet Street.]

### To CORRESPONDENTS.

Communications are received from Dr. Walker, Mr. Simmons, Mr. Bishopp, Mr. Gillespie, Mr. Pulley; and several anonymous signatures, on the subjects of Gout and Vaccination.

